# Supplementary material for: Longitudinal and Multimodal Radiomics Models for Head and Neck Cancer Outcome Prediction
Source: Cancers (Basel). 2023 Jan 21;15(3):673. doi: 10.3390/cancers15030673 (PMC9913206; doi:10.3390/cancers15030673)
Supplement: Supplementary file 1 [file cancers-15-00673-s001.zip › cancers-2103461-supplementary.pdf]

Supplement to: Longitudinal and multimodal radiomics models for head-and-neck cancer outcome prediction

Contents

1 Image acquisition parameters 2

2 Performance of final signature models based on univariate regression feature selection 4

3 Stratification 5

4 Final feature signatures 7

5 Visualization of feature membership in final signatures 9

6 Delta-radiomics: Performance 13

7 Delta-radiomics: Signatures 15

8 Feature differences between cohorts 17

9 Prediction differences between cohorts 19

1 Image acquisition parameters

Table S1: **Image acquisition parameters (CT):**

Variable	Exploration			Ind. validation		
	W0	W2	W3	W0	W2	W3
Spacing z (mm)						
2	0	1	0	0	0	0
3	9	0	6	0	0	0
5	28	36	31	18	18	18
Spacing x,y (mm)						
0.98	9	0	6	0	0	0
1.37	28	37	31	18	18	18
Tube voltage (kV)						
120	35	37	37	18	18	18
unknown	2	0	0	0	0	0
Reconstruction kernel						
B10s	1	0	0	0	0	0
B19f	0	9	1	0	0	0
B20f	25	28	30	16	18	18
B20s	0	0	0	2	0	0
B31f	9	0	6	0	0	0
unknown	2	0	0	0	0	0
Exposure time (ms)						
420	0	28	15	0	18	18
500	34	9	22	16	0	0
750	1	0	0	2	0	0
unknown	2	0	0	0	0	0

Abbreviations: Ind., Independent

Table S2: **Image acquisition parameters (FDG-PET):**

Variable	Exploration		Ind. validation	
	W0	W3	W0	W3
Spacing z (mm)				
3	0	3	0	0
5	37	34	18	18
Spacing x,y (mm)				
0.98	0	3	0	0
1.37	37	6	0	0
4.06	0	28	18	18
Kernel				
Gaussian FWHM=5	37	37	18	18
Reconstruction method				
OSEM2D 6i4s	37	37	18	18
Median uptake time (range)	84.1 (60.9 - 177.8)	82.6 (59.3 - 121.8)	86.4 (64.9 - 119.0)	74.1 (53.5 - 157.7)

Abbreviations: Ind., Independent

2 Performance of final signature models based on univariate regression feature selection

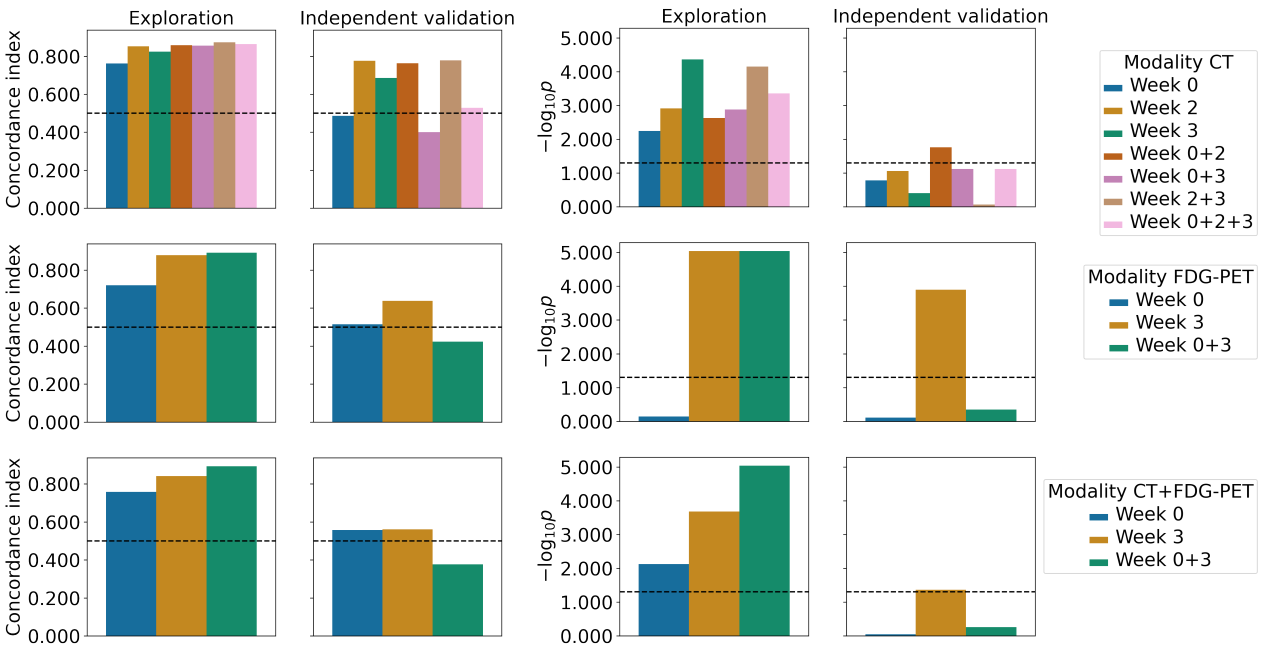

(a) Concordance index

(b) Logrank p-value, logscaled

Figure S1: Performance of the final signature models. The final signature was determined from the univariate regression feature importance scores of all bootstraps from the cross-validation. Values above the dashed line indicate non-random predictions and statistically significant stratifications, respectively.

3 Stratification

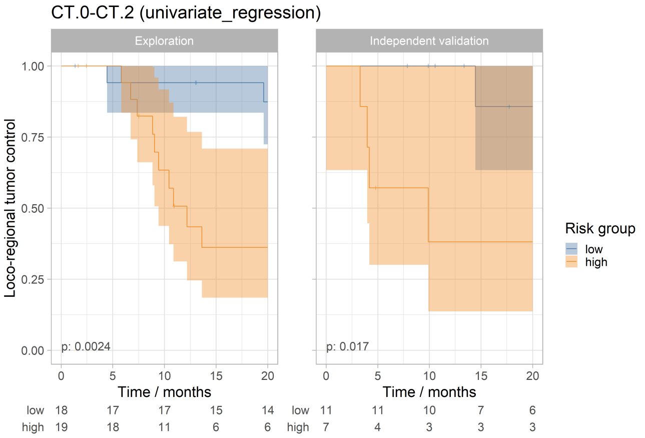

(a) CT W0+2 from univ.

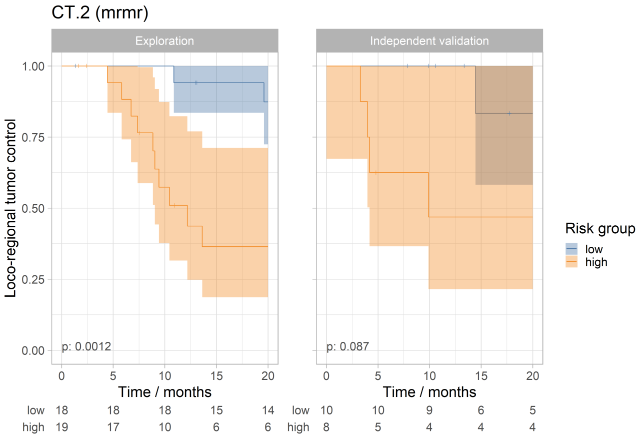

(b) CT W2 from MRMR

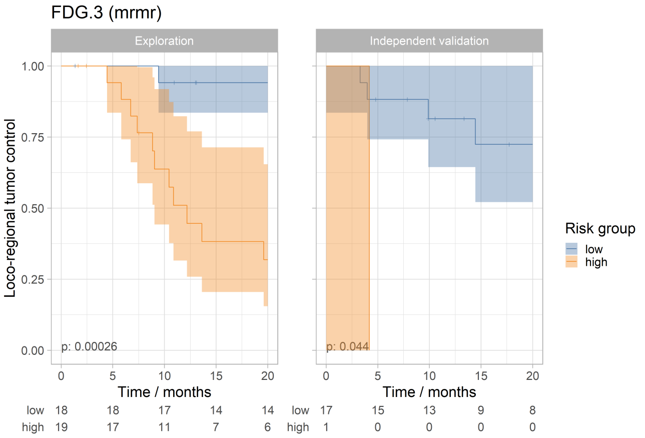

(c) FDG W3 from MRMR

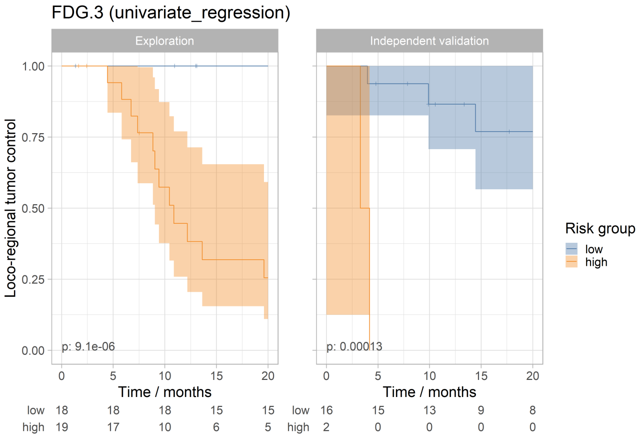

(d) FDG W3 from univ.

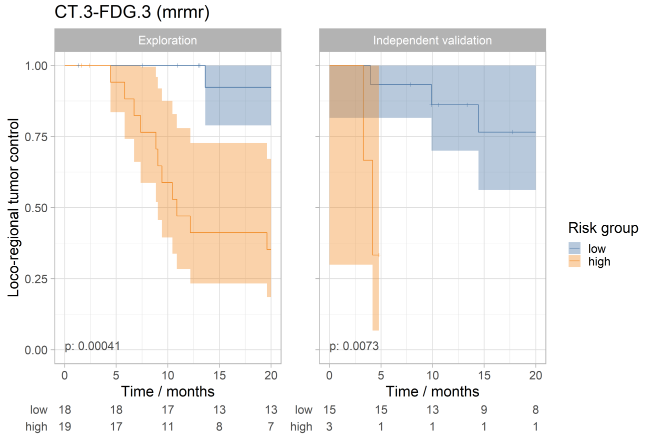

(e) CT+FDG W3 from MRMR

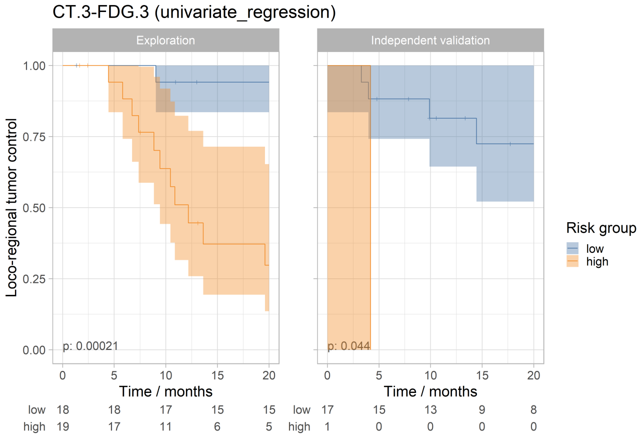

(f) CT+FDG W3 from univ.

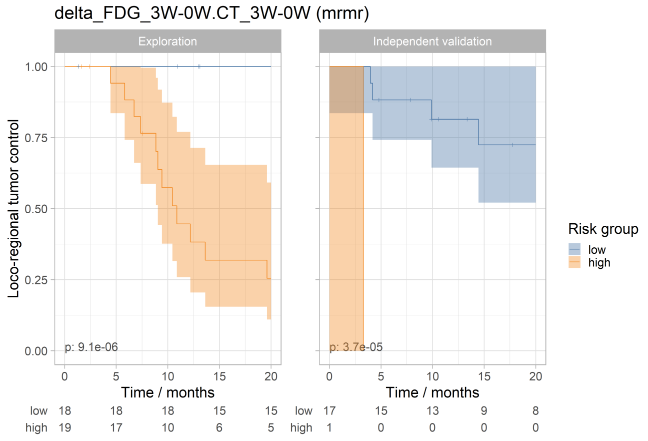

(g) Delta-CT+FDG W3-0 from MRMR

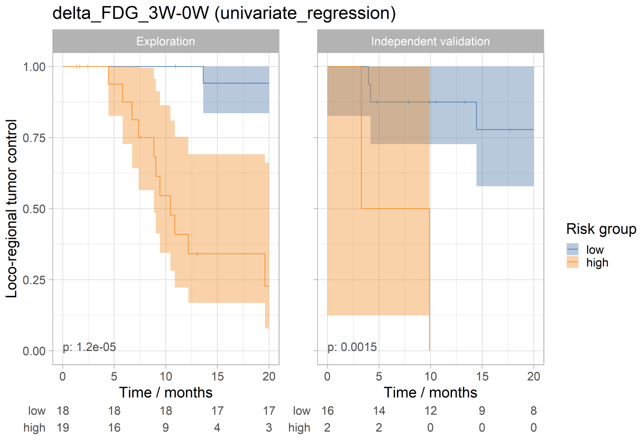

(h) Delta-FDG W3-0 from univ.

Figure S2: Stratifications created by some final signature models. The cutoff was determined as the median prediction on the exploratory cohort.

4 Final feature signatures

Table S3: **Final signature features (CT):** Values in parenthesis denote occurence among the top five features in percent

Experiment	Feature selection	Feature 1	Feature 2	Feature 3	Feature 4
Week 0	MRMR<br>univariate	CT_0W_morph_pca_elongation (29)<br>CT_0W_szm_zs_entr_3d_fbs_w12_0 (33)	CT_0W_ih_mode_fbs_w12_0 (26)<br>CT_0W_szm_lzlge_3d_fbs_w12_0 (31)	CT_0W_rlm_srhge_3d_v_mrg_fbs_w12_0 (21)<br>CT_0W_ngl_ldhge_d1_a0_0_3d_fbs_w12_0 (23)	CT_0W_dzm_zd_var_3d_fbs_w12_0 (19)<br>CT_0W_rlm_srhge_3d_v_mrg_fbs_w12_0 (23)
Week 2	MRMR<br>univariate	CT_2W_cm_info_corr2_d1_3d_v_mrg_fbs_w12_0 (48)<br>CT_2W_cm_info_corr2_d1_3d_v_mrg_fbs_w12_0 (55)	CT_2W_stat_kurt (47)<br>CT_2W_stat_kurt (55)	CT_2W_morph_vol_dens_aee (38)<br>CT_2W_dzm_sde_3d_fbs_w12_0 (33)	CT_2W_dzm_sde_3d_fbs_w12_0 (32)<br>CT_2W_morph_vol_dens_aee (32)
Week 3	MRMR<br>univariate	CT_3W_morph_area_dens_aee (32)<br>CT_3W_szm_zs_entr_3d_fbs_w12_0 (37)	CT_3W_morph_vol_dens_aee (31)<br>CT_3W_morph_vol_dens_aee (35)	CT_3W_morph_pca_flatness (30)<br>CT_3W_morph_area_dens_aee (31)	CT_3W_szm_zs_entr_3d_fbs_w12_0 (25)<br>CT_3W_dzm_lde_3d_fbs_w12_0 (28)
Week 0+2	MRMR<br>univariate	CT_2W_stat_kurt (48)<br>CT_2W_stat_kurt (53)	CT_2W_cm_info_corr2_d1_3d_v_mrg_fbs_w12_0 (23)<br>CT_2W_cm_info_corr2_d1_3d_v_mrg_fbs_w12_0 (29)	CT_0W_morph_pca_elongation (23)<br>CT_2W_dzm_lde_3d_fbs_w12_0 (20)	CT_2W_morph_vol_dens_aee (22)<br>CT_2W_morph_vol_dens_aee (19)
Week 0+3	MRMR<br>univariate	CT_3W_morph_vol_dens_aee (36)<br>CT_3W_morph_vol_dens_aee (30)	CT_0W_morph_pca_elongation (28)<br>CT_0W_ngl_ldhge_d1_a0_0_3d_fbs_w12_0 (24)	CT_0W_rlm_srhge_3d_v_mrg_fbs_w12_0 (21)<br>CT_0W_rlm_srhge_3d_v_mrg_fbs_w12_0 (22)	CT_3W_morph_pca_flatness (17)<br>CT_3W_dzm_sde_3d_fbs_w12_0 (20)
Week 2+3	MRMR<br>univariate	CT_2W_stat_kurt (51)<br>CT_2W_stat_kurt (51)	CT_3W_morph_vol_dens_aee (27)<br>CT_3W_morph_vol_dens_aee (29)	CT_2W_dzm_lde_3d_fbs_w12_0 (20)<br>CT_2W_dzm_lde_3d_fbs_w12_0 (26)	CT_2W_cm_info_corr2_d1_3d_v_mrg_fbs_w12_0 (20)<br>CT_2W_cm_info_corr2_d1_3d_v_mrg_fbs_w12_0 (22)
Week 0+2+3	MRMR<br>univariate	CT_2W_stat_kurt (27)<br>CT_2W_stat_kurt (29)	CT_0W_rlm_srhge_3d_v_mrg_fbs_w12_0 (26)<br>CT_3W_morph_vol_dens_aee (24)	CT_3W_morph_vol_dens_aee (24)<br>CT_0W_rlm_srhge_3d_v_mrg_fbs_w12_0 (21)	CT_2W_cm_info_corr2_d1_3d_v_mrg_fbs_w12_0 (18)<br>CT_2W_dzm_lde_3d_fbs_w12_0 (20)

Table S4: **Final signature features (FDG-PET):** Values in parenthesis denote occurence among the top five features in percent

Experiment	Feature selection	Feature 1	Feature 2	Feature 3	Feature 4
Week 0	MRMR<br>univariate	FDG_0W_morph_pca_elongation (38)<br>FDG_0W_ngt_strength_3d_fbs_w0_25 (34)	FDG_0W_morph_vol_dens_aee (30)<br>FDG_0W_morph_pca_flatness (30)	FDG_0W_morph_pca_flatness (29)<br>FDG_0W_morph_pca_elongation (24)	FDG_0W_stat_min (25)<br>FDG_0W_morph_pca_min_axis (24)
Week 3	MRMR<br>univariate	FDG_3W_dzm_sde_3d_fbs_w0_25 (59)<br>FDG_3W_dzm_sde_3d_fbs_w0_25 (78)	FDG_3W_morph_vol_dens_aee (38)<br>FDG_3W_ngl_dcnu_d1_a0_0_3d_fbs_w0_25 (31)	FDG_3W_ngt_strength_3d_fbs_w0_25 (30)<br>FDG_3W_szm_zsnu_3d_fbs_w0_25 (25)	FDG_3W_morph_pca_flatness (25)<br>FDG_3W_ih_entropy_fbs_w0_25 (25)
Week 0+3	MRMR<br>univariate	FDG_3W_dzm_sde_3d_fbs_w0_25 (51)<br>FDG_3W_dzm_sde_3d_fbs_w0_25 (60)	FDG_0W_stat_min (38)<br>FDG_3W_szm_zsnu_3d_fbs_w0_25 (35)	FDG_3W_ngt_strength_3d_fbs_w0_25 (20)<br>FDG_3W_stat_energy (34)	FDG_0W_morph_pca_elongation (18)<br>FDG_3W_cm_clust_shade_d1_3d_v_mrg_fbs_w0_25 (18)

Table S5: **Final signature features (CT+FDG-PET):** Values in parenthesis denote occurence among the top five features in percent

Experiment	Feature selection	Feature 1	Feature 2	Feature 3	Feature 4
Week 0	MRMR	FDG_0W_morph_pca_elongation (33)	FDG_0W_stat_min (22)	FDG_0W_ih_min_fbs_w0_25 (18)	CT_0W_ih_mode_fbs_w12_0 (14)
	univariate	CT_0W_szm_zs_entr_3d_fbs_w12_0 (26)	CT_0W_szm_lzlge_3d_fbs_w12_0 (23)	FDG_0W_morph_pca_elongation (22)	CT_0W_cm_inv_diff_norm_d1_3d_v_mrg_fbs_w12_0 (18)
Week 3	MRMR	FDG_3W_dzm_sde_3d_fbs_w0_25 (55)	CT_3W_morph_vol_dens_aee (23)	CT_3W_morph_area_dens_conv_hull (14)	CT_3W_morph_pca_flatness (14)
	univariate	FDG_3W_dzm_sde_3d_fbs_w0_25 (68)	FDG_3W_szm_zsnu_3d_fbs_w0_25 (31)	FDG_3W_ngl_dcnu_d1_a0_0_3d_fbs_w0_25 (25)	FDG_3W_stat_energy (23)
Week 0+3	MRMR	FDG_3W_dzm_sde_3d_fbs_w0_25 (41)	FDG_0W_morph_pca_elongation (18)	FDG_0W_stat_min (17)	FDG_3W_szm_zsnu_3d_fbs_w0_25 (15)
	univariate	FDG_3W_dzm_sde_3d_fbs_w0_25 (44)	FDG_3W_szm_zsnu_3d_fbs_w0_25 (34)	FDG_3W_ih_entropy_fbs_w0_25 (22)	FDG_3W_cm_clust_shade_d1_3d_v_mrg_fbs_w0_25 (15)

5 Visualization of feature membership in final signatures

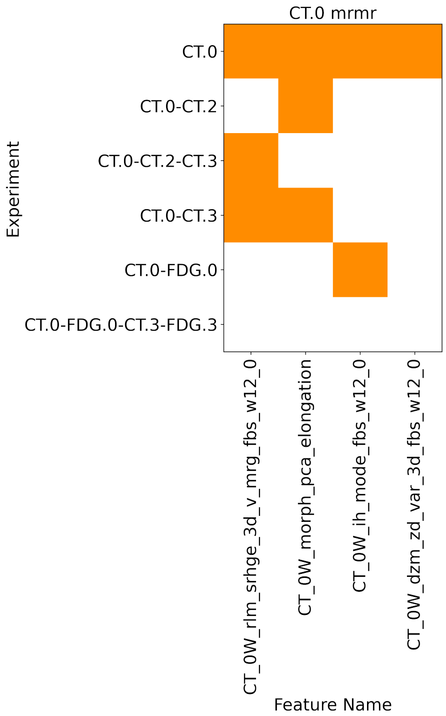

(a) CT0 features

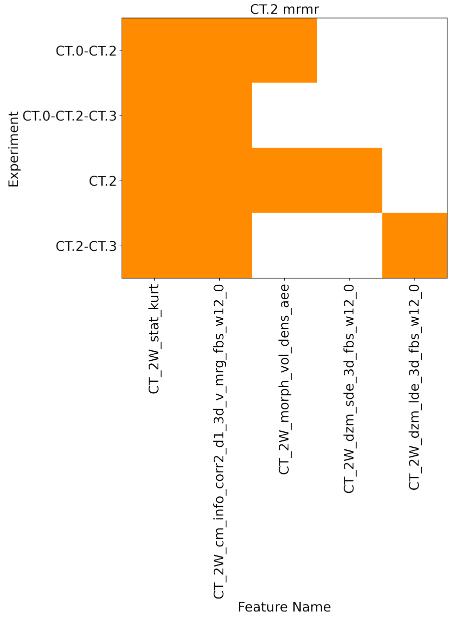

(b) CT2 features

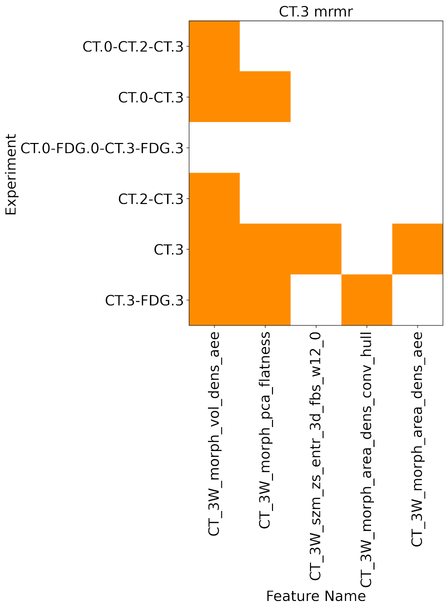

(c) CT3 features

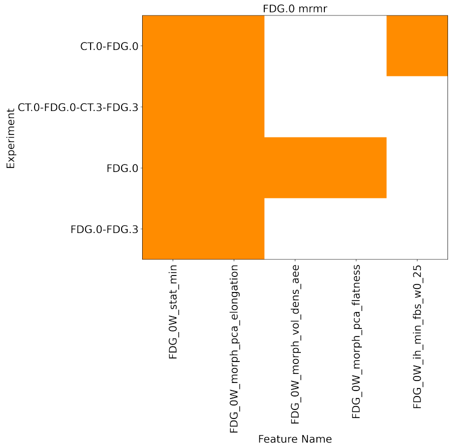

(d) FDG0 features

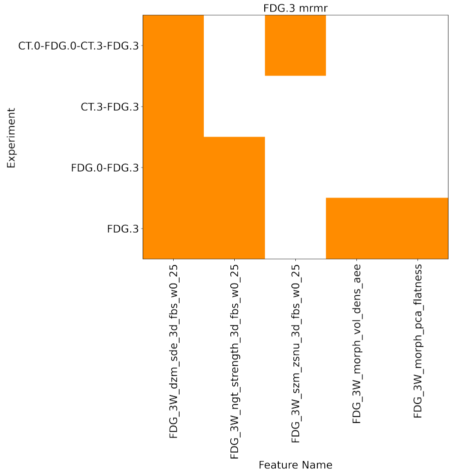

(e) FDG3 features

Figure S3: Selected features by MRMR shown by modality and timepoint. For each modality and timepoint, membership of a feature in an experiment‘s final signature is denoted in orange.

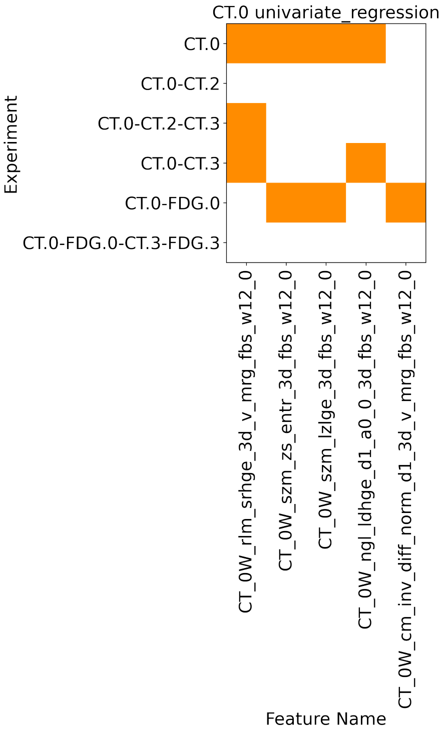

(a) CT0 features

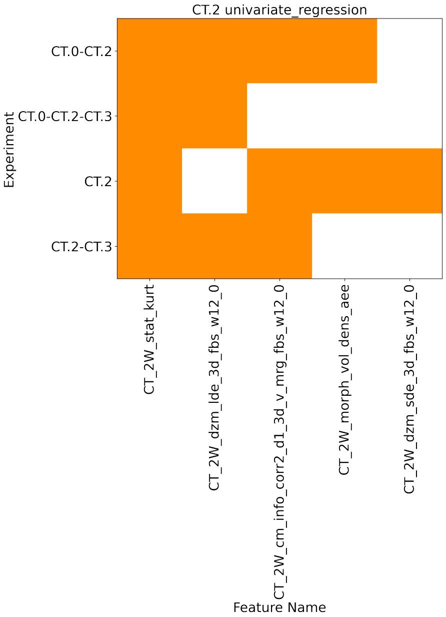

(b) CT2 features

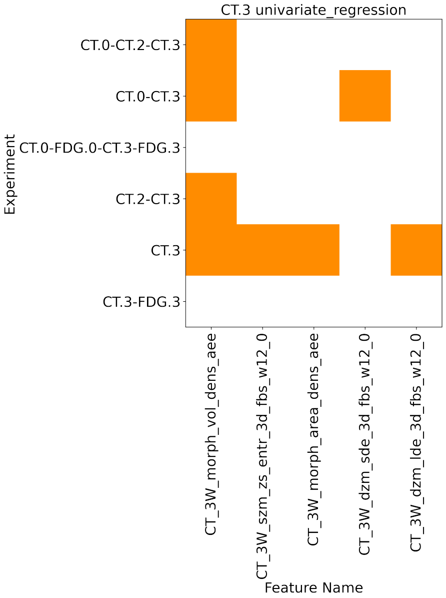

(c) CT3 features

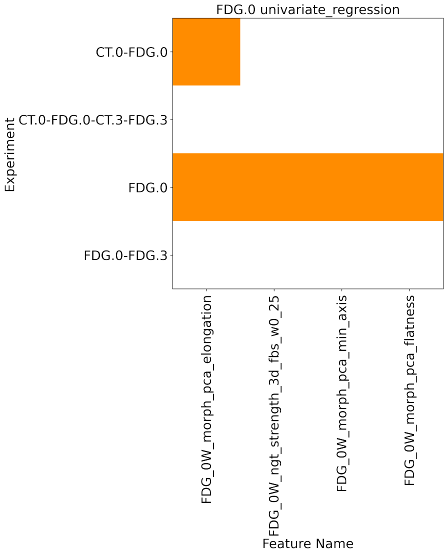

(d) FDG0 features

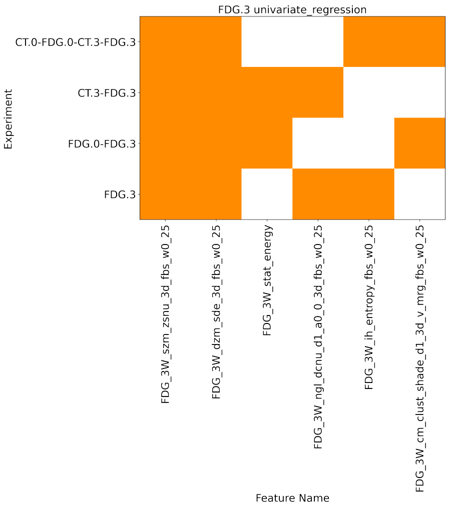

(e) FDG3 features

Figure S4: Selected features by univariate regression shown by modality and timepoint. For each modality and timepoint, membership of a feature in an experiment‘s final signature is denoted in orange.

6 Delta-radiomics: Performance

Table S6: **Final signature model performance (CT):** Values in parenthesis denote 95% confidence intervals based on bootstrapping. Values in bold denote best validation performance. The last column provides the fraction of patients assigned to the low-risk group based on the prediction cutoff. The number of patients in the low-risk group of the exploratory cohort are always 18 out of 37 because the median prediction was used as cutoff.

Experiment	Feature selection	C-index		Log-rank p-value		Patients in low risk group
		Exploration	Ind. validation	Exploration	Ind. validation	Ind. validation
Week 2-0	MRMR	0.79 (0.66 - 0.91)	**0.80 (0.37 - 0.97)**	0.003	0.94	7/18
	univariate	0.76 (0.61 - 0.89)	0.69 (0.30 - 0.91)	0.010	0.86	8/18
Week 3-0	MRMR	0.76 (0.55 - 0.93)	0.42 (0.07 - 0.87)	0.002	0.14	7/18
	univariate	0.76 (0.55 - 0.93)	0.42 (0.07 - 0.87)	0.002	0.14	7/18
Week 3-2	MRMR	0.74 (0.56 - 0.89)	0.64 (0.37 - 0.88)	0.051	NA (all in low-risk group)	18/18
	univariate	0.74 (0.53 - 0.89)	0.58 (0.25 - 0.86)	0.22	0.32	13/18
Week 2-0, 3-0	MRMR	0.82 (0.65 - 0.95)	0.41 (0.13 - 0.71)	0.005	0.94	5/18
	univariate	0.81 (0.64 - 0.96)	0.54 (0.14 - 0.87)	0.005	0.76	5/18
Week 2-0, 3-2	MRMR	0.79 (0.64 - 0.92)	**0.80 (0.49 - 0.97)**	0.054	0.64	6/18
	univariate	0.78 (0.62 - 0.92)	0.71 (0.37 - 0.96)	0.046	0.58	8/18
Week 3-0, 3-2	MRMR	0.83 (0.68 - 0.96)	0.58 (0.20 - 0.92)	0.003	0.27	2/18
	univariate	0.83 (0.68 - 0.96)	0.58 (0.20 - 0.92)	0.003	0.27	2/18
Week 2-0, 3-0, 3-2	MRMR	0.80 (0.63 - 0.94)	0.52 (0.14 - 0.85)	0.011	0.76	5/18
	univariate	0.81 (0.63 - 0.96)	0.47 (0.12 - 0.83)	0.005	0.49	6/18

Abbreviations: C-index, concordance index; MRMR, minimum redundancy - maximum relevance; univariate, univariate Cox regression; Ind., Independent; NA, not available

Table S7: **Final signature model performance (FDG-PET):** Values in parenthesis denote 95% confidence intervals based on bootstrapping. Values in bold denote best validation performance. The last column provides the fraction of patients assigned to the low-risk group based on the prediction cutoff. The number of patients in the low-risk group of the exploratory cohort are always 18 out of 37 because the median prediction was used as cutoff. $*$ indicates statistically significant p-values for the stratification that produced highly imbalanced groups of low and high-risk of loco-regional recurrence.

Experiment	Feature selection	C-index		Log-rank p-value		Patients in low risk group
		Exploration	Ind. validation	Exploration	Ind. validation	Ind. validation
Week 3-0	MRMR	0.91 (0.78 - 0.97)	0.54 (0.09 - 0.93)	< 0.001	NA (all in low-risk group)	18/18
	univariate	0.90 (0.80 - 0.96)	**0.73 (0.35 - 1.0)**	< 0.001	**0.002***	16/18

Abbreviations: C-index, concordance index; MRMR, minimum redundancy - maximum relevance; univariate, univariate Cox regression; Ind., Independent; NA, not available

Table S8: **Final signature model performance (CT+FDG-PET):** Values in parenthesis denote 95% confidence intervals based on bootstrapping. Values in bold denote best validation performance. The last column provides the fraction of patients assigned to the low-risk group based on the prediction cutoff. The number of patients in the low-risk group of the exploratory cohort are always 18 out of 37 because the median prediction was used as cutoff. $*$ indicates statistically significant p-values for the stratification that produced highly imbalanced groups of low and high-risk of loco-regional recurrence.

Experiment	Feature selection	C-index		Log-rank p-value		Patients in low risk group
		Exploration	Ind. validation	Exploration	Ind. validation	Ind. validation
Week 3-0	MRMR	0.89 (0.79 - 0.95)	**0.67 (0.15 - 0.98)**	< 0.001	$< 0.001^*$	17/18
	univariate	0.89 (0.79 - 0.96)	0.48 (0.20 - 0.93)	< 0.001	0.27	17/18

Abbreviations: C-index, concordance index; MRMR, minimum redundancy - maximum relevance; univariate, univariate Cox regression; Ind., Independent

7 Delta-radiomics: Signatures

Table S9: **Final signature features (CT):** Values in parenthesis denote occurence among the top five features in percent

Experiment	Feature selection	Feature 1	Feature 2	Feature 3	Feature 4
Week 2-0	MRMR<br>univariate	CT_2W_0W_szm_zs_entr_3d_fbs_w12_0 (29)<br>CT_2W_0W_szm_zs_entr_3d_fbs_w12_0 (35)	CT_2W_0W_dzm_zdnu_norm_3d_fbs_w12_0 (25)<br>CT_2W_0W_cm_info_corr2_d1_3d_v_mrg_fbs_w12_0 (27)	CT_2W_0W_cm_info_corr2_d1_3d_v_mrg_fbs_w12_0 (23)<br>CT_2W_0W_dzm_ldhge_3d_fbs_w12_0 (26)	CT_2W_0W_loc_peak_loc (18)<br>CT_2W_0W_ngl_hdlge_d1_a0_0_3d_fbs_w12_0 (24)
Week 3-0	MRMR<br>univariate	CT_3W_0W_morph_area_dens_aee (44)<br>CT_3W_0W_szm_lzlge_3d_fbs_w12_0 (39)	CT_3W_0W_ivh_i50 (38)<br>CT_3W_0W_ivh_i50 (30)	CT_3W_0W_szm_lzlge_3d_fbs_w12_0 (32)<br>CT_3W_0W_morph_area_dens_aee (28)	CT_3W_0W_rlm_rlnu_3d_v_mrg_fbs_w12_0 (18)<br>CT_3W_0W_rlm_rlnu_3d_v_mrg_fbs_w12_0 (21)
Week 3-2	MRMR<br>univariate	CT_3W_2W_morph_area_dens_aee (45)<br>CT_3W_2W_morph_area_dens_aee (26)	CT_3W_2W_rlm_rl_entr_3d_v_mrg_fbs_w12_0 (21)<br>CT_3W_2W_ih_p10_fbs_w12_0 (23)	CT_3W_2W_morph_pca_least_axis (19)<br>CT_3W_2W_rlm_rl_entr_3d_v_mrg_fbs_w12_0 (22)	CT_3W_2W_morph_com (18)<br>CT_3W_2W_stat_kurt (21)
Week 2-0, 3-0	MRMR<br>univariate	CT_3W_0W_morph_area_dens_aee (30)<br>CT_3W_0W_szm_lzlge_3d_fbs_w12_0 (25)	CT_3W_0W_ivh_i50 (30)<br>CT_3W_0W_ivh_i50 (25)	CT_2W_0W_dzm_zdnu_norm_3d_fbs_w12_0 (17)<br>CT_2W_0W_szm_zs_entr_3d_fbs_w12_0 (19)	CT_2W_0W_cm_info_corr2_d1_3d_v_mrg_fbs_w12_0 (17)<br>CT_2W_0W_cm_info_corr2_d1_3d_v_mrg_fbs_w12_0 (18)
Week 2-0, 3-2	MRMR<br>univariate	CT_3W_2W_morph_area_dens_aee (31)<br>CT_2W_0W_dzm_lde_3d_fbs_w12_0 (22)	CT_2W_0W_dzm_zdnu_norm_3d_fbs_w12_0 (19)<br>CT_2W_0W_szm_zs_entr_3d_fbs_w12_0 (21)	CT_2W_0W_cm_info_corr2_d1_3d_v_mrg_fbs_w12_0 (16)<br>CT_2W_0W_szm_lzlge_3d_fbs_w12_0 (21)	CT_2W_0W_szm_lzlge_3d_fbs_w12_0 (13)<br>CT_2W_0W_ngl_hdlge_d1_a0_0_3d_fbs_w12_0 (20)
Week 3-0, 3-2	MRMR<br>univariate	CT_3W_0W_morph_vol_dens_aee (26)<br>CT_3W_0W_szm_lzlge_3d_fbs_w12_0 (26)	CT_3W_0W_ivh_i50 (25)<br>CT_3W_0W_ivh_i50 (24)	CT_3W_0W_morph_area_dens_aee (23)<br>CT_3W_0W_morph_vol_dens_aee (19)	CT_3W_0W_szm_lzlge_3d_fbs_w12_0 (20)<br>CT_3W_0W_morph_area_dens_aee (18)
Week 2-0, 3-0, 3-2	MRMR<br>univariate	CT_3W_0W_ivh_i50 (30)<br>CT_3W_0W_ivh_i50 (25)	CT_3W_0W_szm_lzlge_3d_fbs_w12_0 (18)<br>CT_3W_0W_szm_lzlge_3d_fbs_w12_0 (25)	CT_2W_0W_cm_info_corr2_d1_3d_v_mrg_fbs_w12_0 (16)<br>CT_2W_0W_cm_info_corr2_d1_3d_v_mrg_fbs_w12_0 (18)	CT_3W_0W_morph_area_dens_aee (16)<br>CT_2W_0W_dzm_zdnu_norm_3d_fbs_w12_0 (15)

Table S10: **Final signature features (FDG-PET):** Values in parenthesis denote occurence among the top five features in percent

Experiment	Feature selection	Feature 1	Feature 2	Feature 3	Feature 4
Week 3-0	MRMR<br>univariate	FDG_3W_0W_dzm_zdnu_norm_3d_fbs_w0_25 (51)<br>FDG_3W_0W_dzm_zdnu_norm_3d_fbs_w0_25 (50)	FDG_3W_0W_dzm_sde_3d_fbs_w0_25 (37)<br>FDG_3W_0W_dzm_zd_entr_3d_fbs_w0_25 (47)	FDG_3W_0W_ngt_strength_3d_fbs_w0_25 (33)<br>FDG_3W_0W_dzm_sde_3d_fbs_w0_25 (37)	FDG_3W_0W_ih_min_grad_g_fbs_w0_25 (28)<br>FDG_3W_0W_ngt_strength_3d_fbs_w0_25 (25)

Table S11: **Final signature features (CT+FDG-PET):** Values in parenthesis denote occurence among the top five features in percent

Experiment	Feature selection	Feature 1	Feature 2	Feature 3	Feature 4
Week 3-0	MRMR	FDG_3W_0W_dzm_sde_3d_fbs_w0_25 (53)	FDG_3W_0W_dzm_zdnu_norm_3d_fbs_w0_25 (34)	CT_3W_0W_ivh_i50 (31)	CT_3W_0W_morph_area_dens_aee (21)
	univariate	FDG_3W_0W_dzm_sde_3d_fbs_w0_25 (46)	FDG_3W_0W_dzm_zdnu_norm_3d_fbs_w0_25 (31)	FDG_3W_0W_dzm_zd_entr_3d_fbs_w0_25 (29)	CT_3W_0W_ivh_i50 (26)

8 Feature differences between cohorts

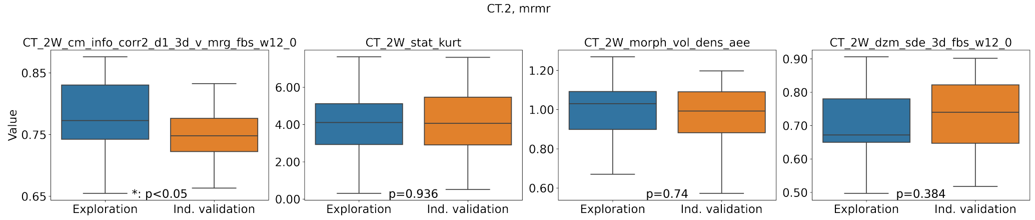

(a) CT W2 from MRMR

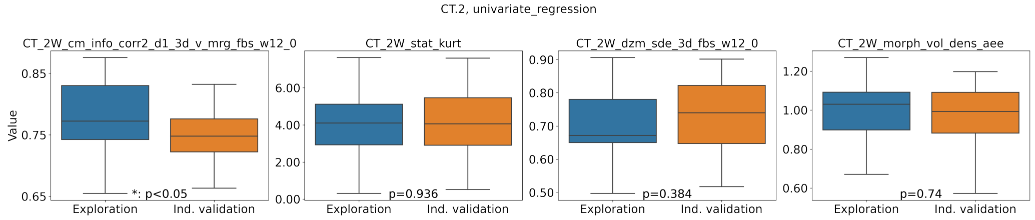

(b) CT W2 from univ.

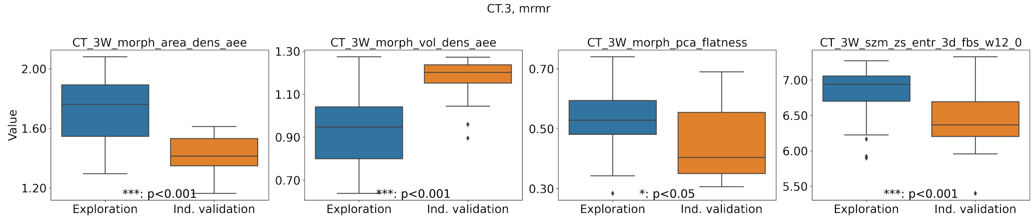

(c) CT W3 from MRMR

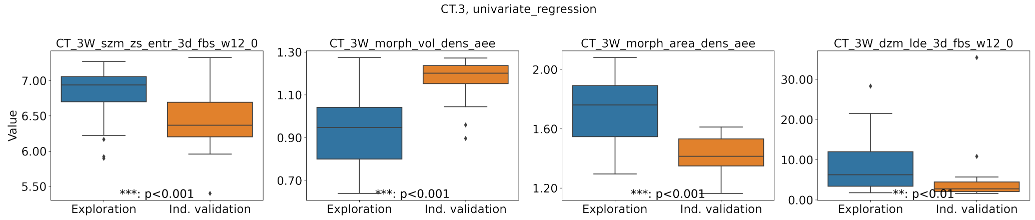

(d) CT W3 from univ.

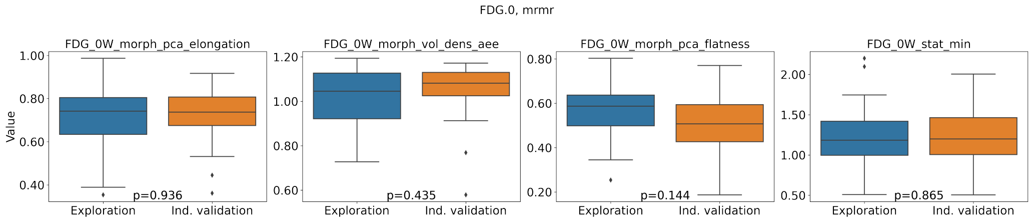

(e) FDG W0 from MRMR

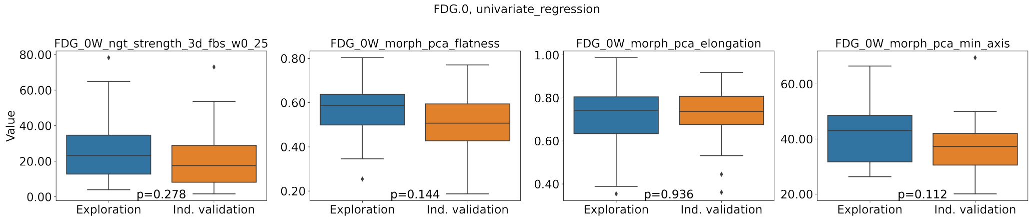

(f) FDG W0 from univ.

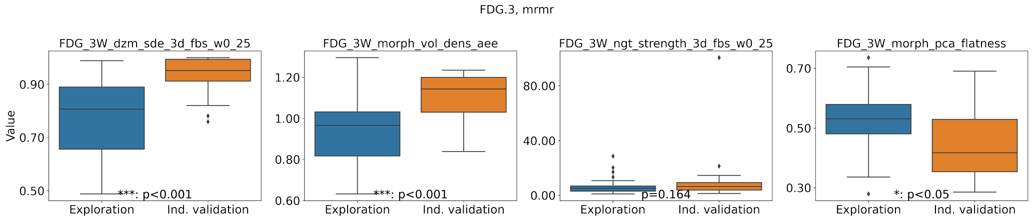

(g) FDG W3 from MRMR

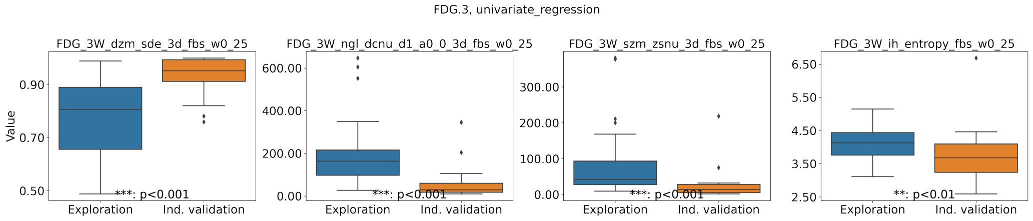

(h) FDG W3 from univ.

Figure S5: Differences between the features of the final signatures between the exploration and independent validation cohort. Testing for significant differences was performed using a Mann-Whitney U-test for which the p-values are shown. $*$ indicates a p-value below 0.05, $**$ below 0.01 and $***$ below 0.001.

9 Prediction differences between cohorts

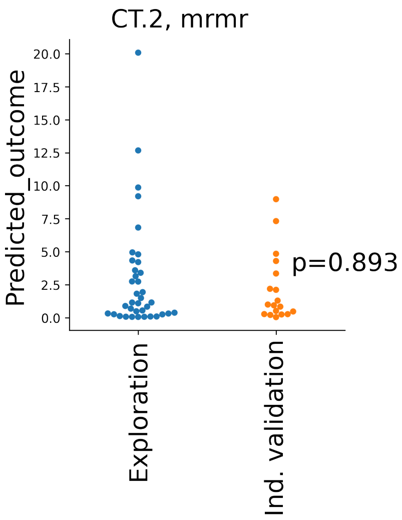

(a) CT W2 from MRMR

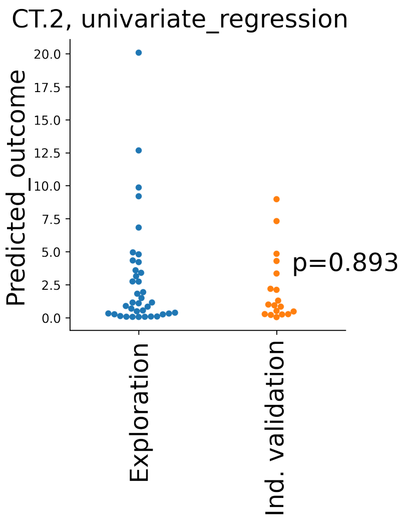

(b) CT W2 from univ.

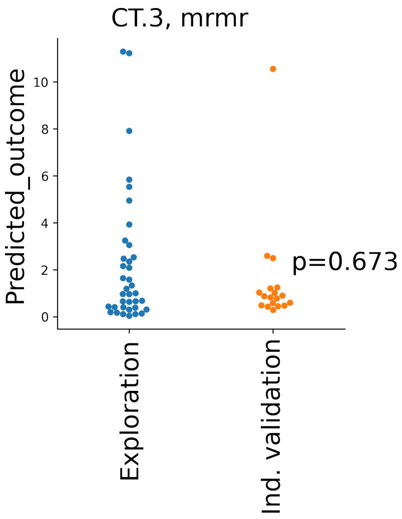

(c) CT W3 from MRMR

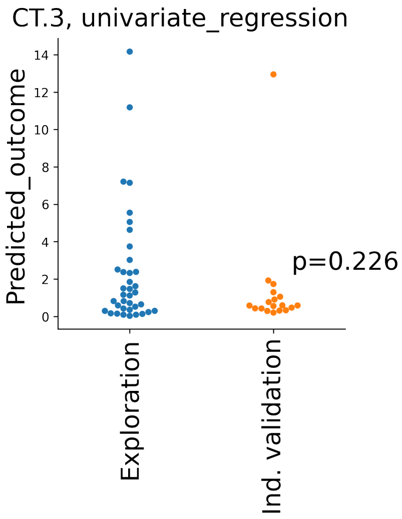

(d) CT W3 from univ.

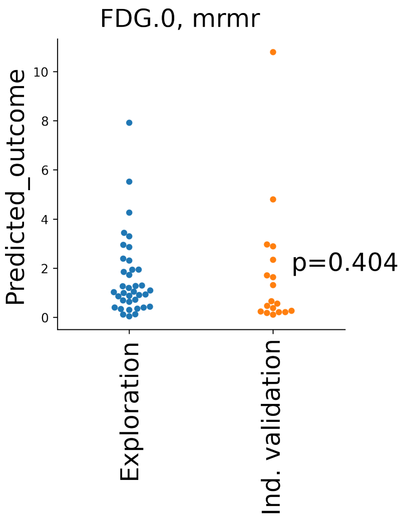

(e) FDG W0 from MRMR

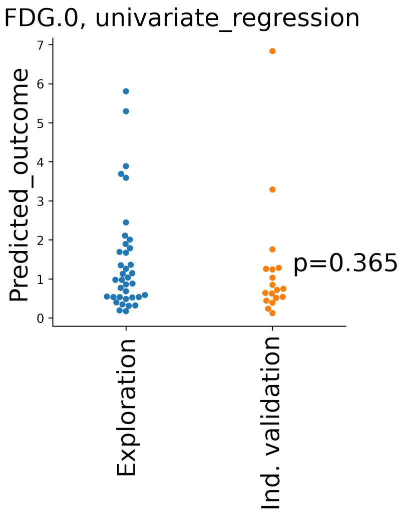

(f) FDG W0 from univ.

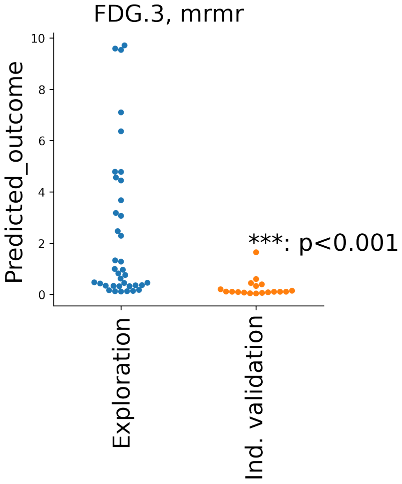

(g) FDG W3 from MRMR

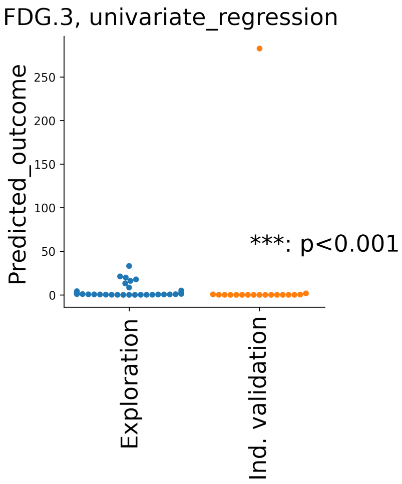

(h) FDG W3 from univ.

Figure S6: Differences between the predictions between the exploration and independent validation cohort. Testing for significant differences was performed using a Mann-Whitney U-test for which the p-values are shown. $*$ indicates a p-value below 0.05, $**$ below 0.01 and $* * *$ below 0.001.
